# Clinical analysis of 18 cases of intraumbilical vascular thrombosis

**DOI:** 10.3389/fsurg.2025.1527353

**Published:** 2025-07-16

**Authors:** Guoliang Tang, Yuting Ye, Yinjiao Li, Jing Xu, Cuixiang Zhou, Yuying Chen, Jun Zhou

**Affiliations:** ^1^ Department of Obstetrics, Shenzhen People’s Hospital, 2nd Clinical Medical College of Jinan University, 1st Affiliated Hospital of South University of Science and Technology, Shenzhen, Guangdong, China; ^2^ Department of Pathology, Shenzhen People’s Hospital, 2nd Clinical Medical College of Jinan University, 1st Affiliated Hospital of South University of Science and Technology, Shenzhen, Guangdong, China

**Keywords:** intraumbilical vascular thrombosis, meconium-stained amniotic fluid, abnormal fetal heart monitoring, intrauterine fetal death, pregnancy outcomes

## Abstract

**Objective:**

This study aimed to assess the clinical characteristics and perinatal outcomes of intraumbilical vascular thrombosis, with the objective of improving diagnostic accuracy and developing better management strategies to enhance perinatal outcomes.

**Methods:**

A retrospective analysis was conducted on 18 cases of intraumbilical vascular thrombosis diagnosed at the Obstetrics Department of the First Affiliated Hospital of Southern University of Science and Technology (Shenzhen, China) between January 2017 and December 2022. Data collected included maternal demographics, details of delivery, pregnancy outcomes, and pathological findings from the placenta and umbilical cord.

**Results:**

Intraumbilical vascular thrombosis was difficult to detect during routine prenatal examinations. Only 3 (16.7%) cases were identified through prenatal ultrasound, while the majority were diagnosed either during labor or through postpartum pathological examination. This condition was associated with several adverse perinatal outcomes, including intrauterine fetal death (4 cases, 22.2%), fetal distress, neonatal brain injury, hypoxic-ischemic encephalopathy, and aspiration pneumonia.

**Conclusion:**

Intraumbilical vascular thrombosis is a rare yet serious condition that increases the risk of fetal and perinatal complications. Early detection remains challenging, highlighting the need for comprehensive assessments involving prenatal ultrasound, fetal heart rate monitoring, amniotic fluid analysis, and infection screening. Prompt intervention, including timely termination of pregnancy when necessary, is critical to minimizing adverse outcomes.

## Introduction

The umbilical cord serves as a vital link between the fetus and the placenta, consisting of two umbilical arteries (UAs) and one umbilical vein (UV), which are responsible for nutrient transport, gas exchange, and waste elimination. Thrombosis of these intraumbilical vessels, umbilical artery thrombosis (UAT) and umbilical vein thrombosis (UVT), is a rare but clinically significant condition that can compromise fetal well-being.

The incidence of intraumbilical vascular thrombosis has been reported to range from 0.0025% to 0.045% of all pregnancies, with UVT being more commonly observed than UAT (1–3). These thrombotic events are associated with serious adverse perinatal outcomes, including intrauterine growth restriction (IUGR), preterm delivery, stillbirth, and neonatal death (4–6). UVT, in particular, is strongly correlated with poor fetal prognosis due to the central role of the UV in oxygenated blood delivery. The pathophysiology of UVT is believed to involve Virchow's triad: venous stasis, endothelial injury, and hypercoagulability. Factors, such as excessive cord length, hypercoiled cords, marginal or velamentous insertion, abnormal Wharton's jelly composition, and mechanical compression from knots or torsion may predispose the umbilical vessels to thrombosis (7–9). In some cases, maternal conditions like thrombophilia, diabetes, or infection have also been implicated in promoting a prothrombotic intrauterine environment (10, 11).

Despite its clinical significance, the prenatal diagnosis of umbilical vascular thrombosis remains challenging. While color Doppler ultrasound can sometimes detect vessel dilation, altered flow, or echogenic intraluminal material, the sensitivity of these findings is low, and most cases are only identified postpartum through pathological examination. Moreover, current diagnostic criteria for UVT are not standardized, and distinctions between thrombus formation, postmortem clotting, and physiologic vascular changes can be difficult to ascertain histologically. Some studies have explored histopathologic timing using markers such as hemosiderin deposition or inflammatory cell infiltration, but these methods have yet to be validated for routine clinical use (12–14). Therefore, clinicians must rely on indirect signs, such as abnormal fetal heart patterns, decreased fetal movement (FM), or unexplained intrauterine demise, to raise suspicion for this condition.

In this study, the clinical characteristics and perinatal outcomes of 18 cases of intraumbilical vascular thrombosis diagnosed in our obstetrics department were retrospectively analyzed. The objective was to improve the understanding of this rare condition and contribute to the optimization of prenatal monitoring and perinatal care strategies. Specifically, this study aimed to: (1) identify the clinical and histopathological features associated with UVT and UAT; (2) evaluate the diagnostic challenges and limitations of current prenatal imaging modalities; (3) assess the perinatal outcomes associated with intraumbilical vascular thrombosis; and (4) explore potential gaps in clinical management to inform future strategies for early detection and intervention.

## Materials and methods

### Data sources

A retrospective analysis was conducted on cases of umbilical vessel thrombosis confirmed by macroscopic observation during childbirth and/or postpartum placental umbilical cord pathology in Obstetrics Department of the First Affiliated Hospital of Southern University of Science and Technology (Shenzhen, China) between January 2017 and December 2022. A total of 18 cases were included in the analysis. The diagnosis of umbilical vessel thrombosis was on the basis of the visual examination of the umbilical cord during childbirth, revealing thrombus in the lumen of either the two UAs or one UV. This diagnosis was further confirmed by a postpartum placental pathology report, indicating thrombus formation in the UA and/or UV. This study was approved by the First Affiliated Hospital of Southern University of Science and Technology, and all patients provided informed consent prior to their enrollment.

### Data analysis

Pregnant participants’ baseline demographic and clinical information: Pregnant participants’ age, gravidity, history of adverse pregnancy outcomes, pregnancy complications, and comorbidities were collected.

Pregnancy outcomes: Prenatal ultrasound, antenatal fetal heart monitoring, mode of delivery, gestational age at termination of pregnancy, and outcomes of the fetus or newborn, including birth weight, gender, Apgar score, admission to the neonatal intensive care unit, newborn complications, and growth and development were all assessed.

### Pathological data of the placental umbilical cord

Statistical Analysis: Statistical analysis was performed using SPSS 22.0 software (IBM, Armonk, NY, USA). Descriptive statistics were used to analyze continuous variables, which were expressed as the mean ± standard deviation. Categorical variables were presented as count (percentage).

## Results

General Information: Pregnant participants’ average age was 31.3 ± 3.9 years, and there were 4 cases of advanced maternal age. The BMI ranged from 18.9 to 24 kg/m^2^, with an average of 21.3 ± 2.3 kg/m^2^. There were no cases of malnutrition or obesity. The average number of pregnancies was 2.1 ± 0.8, involving 7 primiparous women and 11 multiparous women. The gestational age at termination of pregnancy ranged from 29 to 40 + 3 weeks, with an average of 36.7 ± 4.2 weeks. Fetal intrauterine death occurred in 22.2% (4/18) of cases. One patient had a history of previous stillbirth of unknown cause and experienced the recurrence of fetal intrauterine death. Moreover, 11 (61.1%) patients experienced complications or comorbidities during pregnancy, including 7 cases of preterm birth, 3 cases of premature rupture of membranes, 1 case of twin-to-twin transfusion syndrome (TTTS), 2 cases of Mediterranean anemia, 1 case of group B streptococcus (GBS), 1 case of hyperthyroidism and premature labor, 1 case of central placenta previa, and 1 case with history of undergoing *in vitro* fertilization and embryo transfer (IVF-ET). None of the abovementioned patients were complicated by gestational diabetes, hypertension, or coagulation disorders. Furthermore, 4 (22.2%) patients underwent amniocentesis or umbilical cord blood sampling during pregnancy, including 2 cases of umbilical cord blood sampling and 2 cases of amniocentesis. Among them, fetal chromosomal abnormalities were identified in 2 patients: one with fetal X-chromosome chimerism and another with a 47, XXY karyotype. However, chromosomal karyotype and microarray analyses did not indicate any abnormalities. Additionally, 5 (27.8%) patients reported decreased fetal movement prior to admission, and one patient was admitted due to abnormal remote fetal heart monitoring.

**Figure 1:**
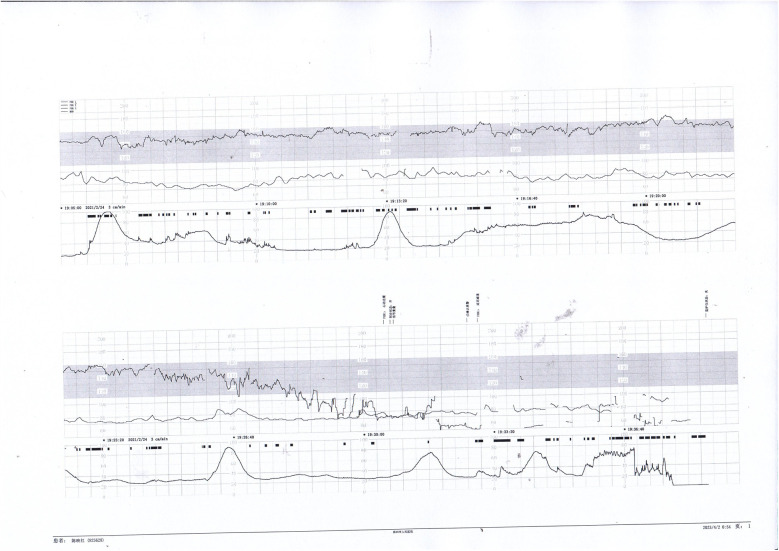
Electronic fetal heart rate monitoring showing variable decelerations and a prolonged deceleration. The infant was later diagnosed with hypoxic-ischemic encephalopathy (HIE), indicating significant intrauterine distress.

**Figure 2:**
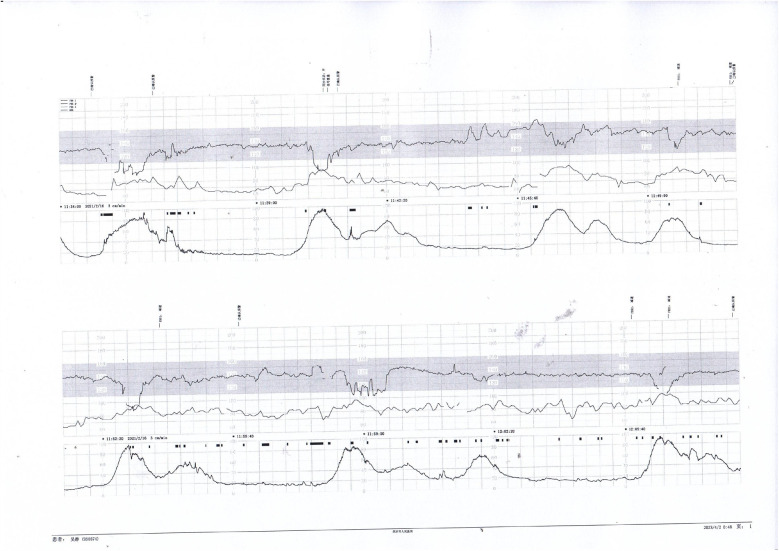
Fetal heart rate tracing demonstrates recurrent early decelerations followed by variable decelerations. The newborn presented with imaging-confirmed brain damage, consistent with sustained intrauterine hypoxia.

**Figure 3:**
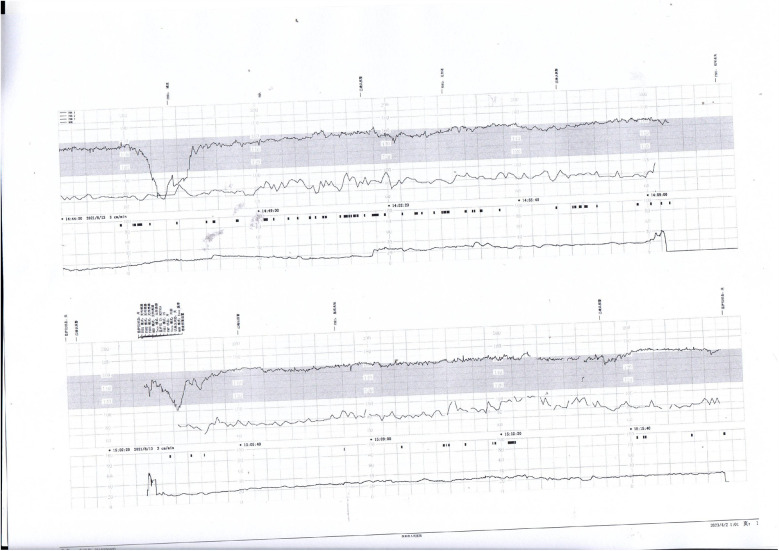
Decreased baseline variability, variable decelerations, and late decelerations were recorded. The infant was diagnosed with intracranial hemorrhage, likely secondary to severe perinatal asphyxia.

**Figure 4:**
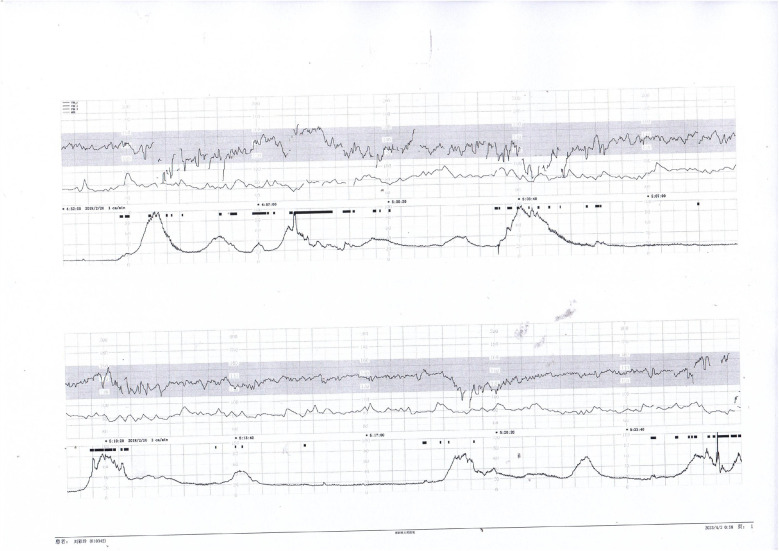
Early and variable decelerations on fetal monitoring in a case of umbilical venous thrombosis. These findings are associated with transient fetal hypoxia.

**Figure 5:**
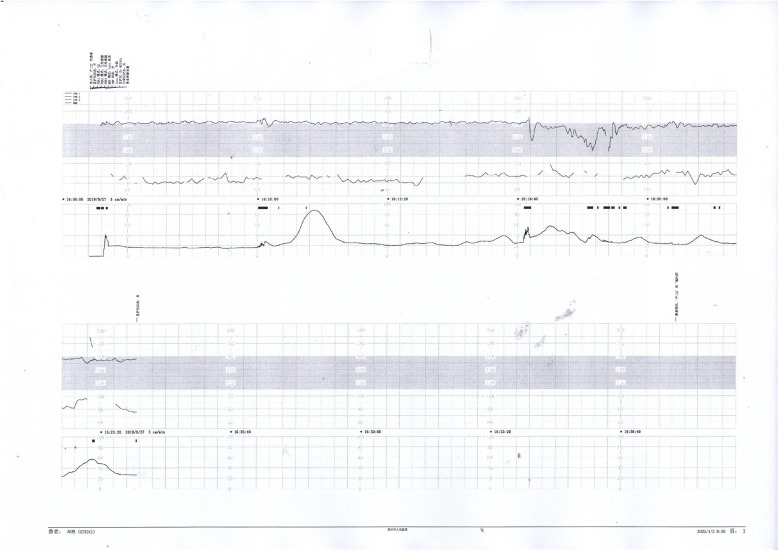
Nonreactive fetal heart pattern with decreased variability and recurrent late decelerations. These parameters suggest fetal compromise and placental insufficiency.

**Figure 6:**
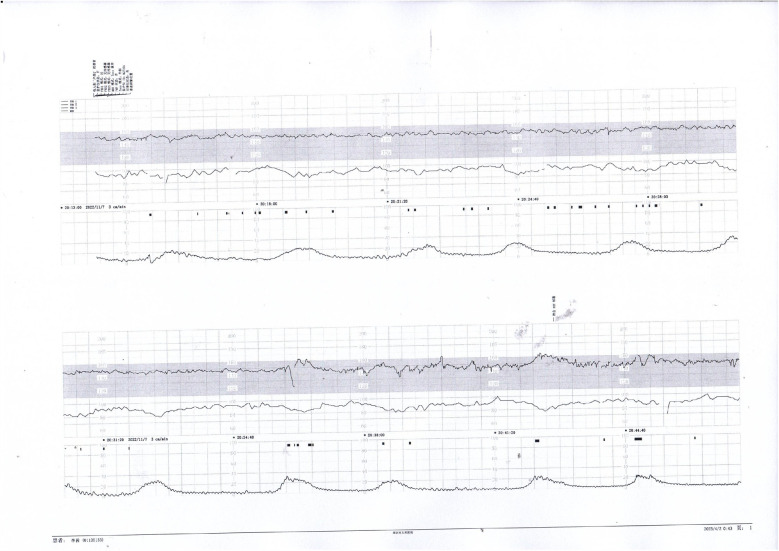
Initial decreased variability on fetal heart monitoring was observed but improved following maternal oxygen inhalation. This case demonstrates a reversible cause of transient fetal hypoxia.

Prenatal Care and Pregnancy Outcomes: Prenatal color Doppler ultrasound suggested possible single umbilical artery (SUA) occlusion in 2 patients and possible UVT with normal umbilical artery S/D ratios in one patient. Fetal heart monitoring was conducted prenatally for all surviving fetuses, revealing abnormal findings in all cases, such as nonreactive patterns, decreased variability, recurrent early decelerations, variable decelerations, late decelerations, or prolonged decelerations. Figures 1–3 illustrate the fetal monitoring patterns for UVT, with corresponding neonatal outcomes in parentheses. Figures 4–6 display the patterns of SUA combined with UVT.

Moreover, 11 (61.1%) patients underwent caesarean section or caesarean delivery to terminate pregnancy, 6 (33.3%) patients received vaginal delivery, and 1 (5.6%) patient required forceps delivery. The average weight of the 4 stillborn fetuses was 1,630 ± 847 g, while the average weight of the 14 newborns was 2,880 ± 579 g. The clinical conditions of the surviving infants are presented in Table 1. They have been followed up to the present, exhibiting notable growth and neurodevelopment.

**Table 1:** Clinical conditions of 14 surviving infants.

Clinical conditions of surviving infants	Number of cases
- Premature infants:	3
- Full-term infants:	11
- Male infants:	6
- Female infants:	8
- Transferred to NICU:	10
- Neonatal asphyxia:	1
- Brain injury:	2
- Intracranial hemorrhage:	2
- HIE (Hypoxic-ischemic encephalopathy):	1
- Respiratory distress syndrome:	2
- Pneumonia:	5
- Acidosis:	5
- Jaundice:	5
- Coagulation dysfunction:	1
- Generalized subcutaneous oedema:	1

To better visualize the relationship between thrombosis type and clinical characteristics or outcomes, an additional table (Table 2) has been included. This table presents individual-level data for all 18 cases, including the type of umbilical thrombosis, fetal or neonatal outcomes, delivery mode, and associated perinatal conditions. No clear correlation was identified between the type of thrombosis and the number or severity of adverse neonatal outcomes. Stillbirth occurred in all three cases with dual UAT involvement, while isolated UVT or UAT cases were more often associated with live births, albeit with varying degrees of neonatal morbidity. Further detailed analysis is warranted in larger cohorts.

**Table 2:** Individual characteristics, thrombosis type, and perinatal outcomes.

Case no.	Thrombosis type	GA and outcome	Delivery mode	Neonatal conditions/findings	Comorbidities/complications	Chromosomal findings	Notes (e.g., FHR, FM ↓)
1	UAT + UVT	33 weeks, Stillbirth	Cesarean	–	Meconium-stained fluid, twisted cord	None	Abnormal FHR, ↓ FM
2	UVT	32 + 4 weeks, Live birth	Vaginal	NICU stay, Low birth weight, TTTS twin	TTTS	None	Abnormal FHR
3	UVT	35 + 5 weeks, Live birth	Cesarean	GBS exposure, neonatal infection	GBS-positive	None	↓ FM, abnormal FHR
4	UAT + UVT	40 + 1 weeks, Stillbirth	Cesarean	–	Twisted cord, meconium fluid	None	History of stillbirth
5	UVT	38 weeks, Live birth	Forceps delivery	Prematurity, jaundice	PROM, anemia	None	↓ FM
6	UVT	39 weeks, Live birth	Cesarean	Normal growth, NICU stay	IVF-ET	47, XXY	FHR ↓
7	UAT	24 + 2, Live birth	Vaginal	Normal	None	None	Normal monitoring
8	UAT + UVT	30 + 5 weeks, Stillbirth	Cesarean	–	Cord around neck, true knot	None	↓ FM, meconium-stained fluid
9	UVT	35 + 2 weeks, Live birth	Cesarean	Hypoxia, NICU stay	Preterm birth	None	↓ FM, abnormal FHR
10	UAT + UVT	30 + 5 weeks, Stillbirth	Cesarean	–	Cord around neck, true knot	None	↓ FM, meconium-stained fluid
11	UAT	36 + 0 weeks, Live birth	Cesarean	Normal	None	None	FHR abnormal, ↓ FM
12	UVT	30 + 4 weeks, Live birth	Vaginal	Bartter syndrome type 4b	Preterm birth, Acute fetal distress	None	History of induced labor at 32 weeks, and intrauterine fetal demise, polyuria, hypokalemia
13	UAT + UVT	33 weeks, Live birth	Cesarean	–	Meconium-stained fluid, twisted cord	None	FHR abnormal, ↓ FM
14	UVT	35 weeks, Live birth	Cesarean	Bartter syndrome	Preterm birth, Weight 2,180g	None	hyponatremia, history of severe polyhydramnios
15	UAT + UVT	40 + 1 weeks, Stillbirth	Cesarean	–	Twisted cord, meconium fluid	None	History of stillbirth
16	UVT	40 weeks, Live birth	Cesarean	NICU stay, no anatomical cord, no umbilical cord torsion	Preterm birth, Weight 2,830 g, healthy	None	No FHR/FM abnormalities, maternal gestational diabetes mellitus
17	UAT	32 + 3 weeks, Live birth	Cesarean	Fetal hypoxia, fetal distress, NICU stay	Chronic nephritis, polyhydramnios	None	FHR abnormal, ↓ FM
18	UVT	31 + 2 weeks, Live birth	Cesarean	–	Preterm birth, Narrowed cord with hyper-coiling	None	FHR abnormal, ↓ FM

Delivery and Postpartum Pathological Data: In this study, the distribution of umbilical cord thrombosis types was as follows: 3 (16.7%) cases of UAT, 9 (50.0%) cases of UVT (Supplementary Figure S6), and 7 (38.9%) cases with both UAT and UVT. Among the mixed cases, 4 cases involved a single UAT with UVT (Supplementary Figures S1, S3–S5), and 3 cases involved two UATs with UVT, all resulting in stillbirth (Supplementary Figure S2). These findings suggest a potential association between dual UAT involvement and poor fetal outcomes. However, this relationship was not consistently found in cases of isolated thrombosis. Table 2 presents case-by-case comparison of thrombosis type and associated clinical characteristics. Additional findings included 7 (38.9%) cases where the umbilical cord was wrapped around the neck once, 5 (27.8%) cases where the umbilical cord was twisted, 4 (22.2%) cases with marginal insertion of the umbilical cord, 2 (11.1%) cases where the umbilical cord was located between the fetal head and the uterine wall, 1 (5.6%) case with a true knot in the umbilical cord, and 1 (5.6%) case with a thinning umbilical cord and a false knot. Meconium-stained amniotic fluid was present in 10 (55.6%) patients, including all 4 stillborn fetuses and 6 live-born infants. Additionally, 1 (5.6%) patient had bloody amniotic fluid combined with a short umbilical cord. Acute chorioamnionitis was found in 5 (27.8%) patients.

## Discussion

The umbilical cord is a flexible structure connecting the fetus to the placenta, typically measuring 30–100 cm in length and containing two umbilical arteries (UAs) and one UV. It is essential for sustaining fetal-placental circulation and supporting fetal growth, development, oxygenation, and survival. Disruption of fetal oxygenation may compromise critical organ systems, particularly the cardiovascular and central nervous systems. Functional or structural abnormalities of the cord can result in fetal or neonatal injury, contributing to elevated fetal and perinatal morbidity and mortality (1–3). Umbilical cord vessel thrombosis is a rare vascular complication, with an estimated incidence of 2.5–4.5 per 10,000 births (4). According to Oliveira et al., it is associated with significantly increased fetal and perinatal morbidity and mortality, although the underlying pathogenesis remains unclear (5). Thrombosis is most often attributed to cord abnormalities that induce mechanical compression and venous dilation (5). Prior research indicates a predilection for involvement of the UV, frequently coexisting with conditions such as abnormal cord insertion, excessive coiling, elongation, constriction, and hypoplasia of Wharton's jelly. Arteriovenous malformations and UV varicosities may also predispose to thrombosis (6). Diagnosis is generally based on ultrasonographic detection of UV dilation exceeding 9 mm or a greater than 50% increase compared to adjacent segments (7).

In this study, the predominant umbilical cord abnormalities included excessive coiling, torsion, marginal insertion, interposition between the fetal head and uterine wall, true knots, thin cords with false knots, and vascular varices. Although maternal hypercoagulable states, endothelial injury, and hyperglycemia have been identified as contributing factors (6), none of the participants exhibited coagulation disorders or abnormal glucose metabolism. UVT has been associated with intrauterine growth restriction, stillbirth, and hypoxic-ischemic encephalopathy. These findings support the need for enhanced fetal surveillance and prompt cesarean delivery in suspected cases to mitigate adverse perinatal outcomes (6, 8).

In the present study, chromosomal abnormalities were identified in two cases following invasive prenatal testing, although microarray and karyotype analyses did not reveal consistent pathogenic findings. These results underscore the complexity of differentiating between structural or vascular umbilical anomalies and potential genetic etiologies of fetal demise or adverse outcomes. Notably, over a quarter of patients (27.8%) reported decreased fetal movement prior to admission. This symptom, though nonspecific, correlated with abnormal fetal heart monitoring in all surviving fetuses, highlighting its potential as an early clinical warning sign for fetal compromise secondary to umbilical vessel thrombosis. The majority of pregnancies were terminated via cesarean section (61.1%), reflecting the urgency often associated with fetal distress linked to umbilical cord thrombosis. Vaginal and assisted deliveries were performed under close surveillance, further supporting the need for individualized delivery planning based on fetal monitoring results and clinical presentation. The average weight of stillborn fetuses (1,630 ± 847 g) was significantly lower than that of live births (2,880 ± 579 g), suggesting a possible association between thrombosis and impaired fetal growth. However, all surviving neonates demonstrated favorable postnatal development during follow-up, reinforcing the importance of timely detection and intervention. A wide range of umbilical cord abnormalities, such as cord entanglement, marginal insertion, torsion, and true knots, were observed, with several cases involving multiple concurrent anomalies. These structural variations may serve as mechanical triggers or predisposing factors for vascular thrombosis, supporting previous hypotheses on cord morphology's role in fetal hemodynamic disruption. Meconium-stained amniotic fluid was present in over half of the cases (55.6%), including all stillbirths. This finding is consistent with fetal distress and hypoxia, and may represent a clinical clue suggestive of compromised cord circulation. One case of bloody amniotic fluid with a short cord further supports the mechanical vulnerability of certain cord configurations. Acute chorioamnionitis was diagnosed in 27.8% of patients, suggesting a potential inflammatory component that may either precede or exacerbate umbilical vessel thrombosis. Although the temporal relationship between infection and thrombosis remains uncertain, inflammation-induced endothelial injury could theoretically contribute to thrombus formation.

In Heifetz SA's analysis of 52 cases of umbilical vessel thrombosis across three patient cohorts, UAT was identified as being more frequently associated with poor fetal outcomes (9). Alhousseini et al. further reported that prenatal identification of a reduction from two umbilical arteries to a SUA correlates with adverse perinatal outcomes, including stillbirth and fetal growth restriction, indicating a generally poor prognosis. Consequently, timely prenatal ultrasound monitoring is crucial for the early detection and management of such vascular anomalies to prevent complications such as stillbirth (4, 5, 10–12). It is important to differentiate prenatal ultrasound findings suggestive of UAT from those of a SUA, a congenital condition characterized by the absence of one UA, most commonly the left. SUA occurs in approximately 0.5%–5% of naturally conceived pregnancies (13). However, prenatal detection remains challenging; in the present study, only three cases were suspected of umbilical vessel thrombosis based on ultrasound findings. Clinical evaluation may also be guided by assessment of the UCI, as proposed by Strong et al. (14). A UCI greater than 0.3 coils/cm (above the 90th percentile) and a coiling pitch less than 3.3 cm indicate excessive coiling and may warrant further investigation. Ultrasound can also aid in the identification of associated abnormalities such as marginal cord insertion and nuchal cord, which may contribute to vascular compromise.

Prenatal diagnosis of umbilical vessel thrombosis remains challenging, with limited diagnostic modalities available. Most cases are detected during labor or in the postpartum period, and only a small proportion are identified prenatally via ultrasound. In this study, several patients experienced decreased fetal movements, and all viable fetuses demonstrated abnormal electronic fetal monitoring (EFM) findings before delivery. One case involved admission due to abnormal remote EFM results, highlighting the importance of educating pregnant women to regularly monitor fetal movements during prenatal care and after hospitalization. Accurate interpretation of EFM is essential for early detection of fetal compromise (15). Key EFM indicators such as reduced baseline variability, recurrent decelerations, prolonged decelerations, or the presence of meconium-stained amniotic fluid should prompt heightened clinical vigilance. Under such circumstances, expedited delivery, typically via cesarean section, should be considered to reduce the risk of fetal morbidity and mortality. When abnormalities are detected via remote monitoring, decisions regarding further evaluation or immediate hospitalization should be guided by the characteristics of the EFM tracing. In cases where the patient is multiparous, labor is imminent, and no severe meconium-stained amniotic fluid is present, vaginal or assisted vaginal delivery may be pursued under close and continuous surveillance.

In this study, the main cause of blood clot formation in the umbilical vessels was UVT, followed by UAT combined with UVT. Among the three cases where both UAT and UVT were present, all incidents were resulted in fetal death. The causal relationship between intrauterine fetal death and thrombosis in the umbilical vessels remains elusive. Several mechanisms have been proposed to explain the pathogenesis of intraumbilical vascular thrombosis, most of which align with Virchow's triad—venous stasis, endothelial injury, and hypercoagulability. Mechanical factors, such as excessive cord length, true knots, torsion, and abnormal insertions (e.g., marginal or velamentous), may induce localized stasis and mechanical injury to the vascular endothelium, facilitating thrombus formation (9). Histopathologic studies suggest that cord compression can cause endothelial denudation and localized inflammation, promoting a prothrombotic environment within the vessel lumen (16). Additionally, abnormalities in Wharton's jelly, such as reduced volume or altered composition, may impair mechanical protection and increase the vulnerability of vessels to torsion and collapse (17). Although none of the mothers in our study had known coagulation disorders, inherited or acquired thrombophilias (e.g., factor V Leiden mutation, antiphospholipid syndrome) have been identified as maternal risk factors for fetal thrombotic vasculopathy in other reports (18, 19). Furthermore, some studies hypothesize that fetal inflammatory responses or metabolic disturbances may independently contribute to endothelial dysfunction and coagulation activation *in utero* (3, 16). While no single mechanism can explain all cases, it is likely that a multifactorial interaction between mechanical, vascular, and hematologic factors contributes to the pathogenesis of IVT. Further research integrating placental pathology, maternal-fetal hemostatic profiles, and cord morphometry is essential to clarify these mechanisms. Bonasoni et al. reported that the neutrophils/macrophages ratio, along with parameters, such as iron-containing hemosiderin deposits, calcium deposition, and angiogenesis, can be used to determine the timing of thrombosis, especially within the first 24 h (20). However, this method is not currently utilized clinically.

Based on our findings, we recommend the following clinical strategies for managing suspected umbilical vascular thrombosis: (1) Routine third-trimester ultrasound assessments should include detailed evaluation of umbilical cord morphology, focusing on coiling index, insertion anomalies, cord entanglement, and vascular dilatation. (2) Any detection of umbilical vessel dilation >9 mm or asymmetric vessel diameters should prompt serial Doppler studies and close fetal surveillance. (3) Abnormal fetal heart rate patterns, particularly persistent reduced variability, recurrent decelerations, and non-reactive tracings, should be regarded as potential signs of compromised umbilical circulation and warrant immediate assessment, including consideration of expedited delivery. (4) For high-risk cases (e.g., with coexisting cord abnormalities or decreased fetal movements), the use of remote fetal heart monitoring and daily fetal movement counting should be standardized during the perinatal period. (5) In facilities with access to fetal magnetic resonance imaging (MRI) or advanced Doppler technologies, these modalities may be considered adjunctive tools to improve diagnostic sensitivity. (6) Finally, standardized postpartum histopathological protocols should be implemented for cases of unexplained stillbirth or neonatal compromise, ensuring the identification of occult umbilical thrombosis and enabling subsequent risk stratification in future pregnancies.

Emerging diagnostic modalities may help overcome the current limitations in the prenatal detection of IVT. High-resolution Doppler imaging, three-dimensional (3D) and four-dimensional (4D) ultrasound, and fetal MRI have shown promise for more detailed evaluations of umbilical cord morphology and blood flow. Advanced Doppler techniques can potentially detect subtle alterations in blood flow velocities and vessel diameters, while quantitative assessments (e.g., automated measurement of the umbilical coiling index) may offer more objective criteria for identifying at-risk cords. In addition, research into circulating biomarkers such as D-dimer levels, endothelial microparticles, and other indicators of fetal coagulation activation could provide non-invasive adjuncts to imaging studies. Integrating these emerging tools with artificial intelligence-based analytics may further refine diagnostic accuracy and risk stratification, ultimately guiding clinicians toward more proactive and tailored management pathways for pregnancies complicated by suspected IVT.

In conclusion, the mechanism of blood clot formation in umbilical vessels remains elusive. Most cases involve venous thrombosis, while arterial thrombosis is less frequent. This condition can lead to an increase in fetal and perinatal morbidity and mortality. Due to the difficulty of prenatal detection, the accurate diagnosis and clinical management of this condition remain unresolved challenges. A comprehensive evaluation based on prenatal ultrasound, fetal heart rate (FHR) monitoring, amniotic fluid characteristics, and the presence of infection is required to timely select the appropriate approaches for terminating the pregnancy and reducing adverse outcomes. This study provides valuable insights into the clinical features, diagnostic limitations, and pathological findings associated with umbilical vessel thrombosis. The relatively high rate of abnormal fetal heart rate patterns and decreased fetal movements preceding delivery highlights the importance of enhanced surveillance in at-risk pregnancies. Although prenatal ultrasound has limited diagnostic sensitivity, combining imaging findings with clinical monitoring may facilitate earlier detection. However, prospective studies are required to assess the long-term effects of IVT in pregnant woman. This would help to identify and analyze high-risk factors, analyze clinical characteristics, and provide strategies to associated pregnancy management. For instance, Zhan et al. study reported that maternal diabetes is a significant risk factor for IVT in pregnant woman with preterm conditions (21). Therefore, identifying specific prenatal risk factors, such as cord abnormalities, meconium-stained amniotic fluid, and decreased fetal movement, can support timely interventions to improve perinatal outcomes. Given the diagnostic challenges and poor prognosis associated with combined arterial and venous thrombosis, further multicenter and prospective studies with larger cohorts and long-term neonatal follow-up are warranted. The development of standardized imaging criteria and biomarker-based screening tools may enhance early recognition and management of this rare but serious condition.

## Data Availability

The raw data supporting the conclusions of this article will be made available by the authors, without undue reservation.

## Supplementary material

Supplementary Figure 1 Gross image of umbilical cord in a live fetus, showing vascular thrombosis with cord torsion. These features suggest mechanical compromise of cord blood flow.

Supplementary Figure 2 Gross pathological specimen of a stillborn fetus showing thrombosis and torsion at the umbilical wheel. The finding supports a mechanical and thrombotic etiology for intrauterine demise.

Supplementary Figure 3 Pathological specimen of an umbilical cord showing a prominent 2 cm × 4 cm protrusion consistent with thrombus. The mass caused focal vascular dilation and distortion.

Supplementary Figure 4 Cross section of the umbilical cord and gross placenta specimen. Visible thrombi are seen within a single umbilical artery (UA) and the umbilical vein (UV), indicating mixed arterial and venous thrombosis.

Supplementary Figure 5 Hematoxylin and eosin (HE) staining of umbilical cord (40× magnification) showing thrombus formation in a single UA and UV. Scale bar = 50 µm.

Supplementary Figure 6 Hematoxylin and eosin (HE) staining (40× magnification) revealing thrombus within the umbilical vein (UV). Scale bar = 50 µm.
