# Supplementary figures and images for: Clinical analysis of 18 cases of intraumbilical vascular thrombosis
Source: Front Surg. 2025 Jul 16;12:1527353. doi: 10.3389/fsurg.2025.1527353 (PMC12309003; doi:10.3389/fsurg.2025.1527353)

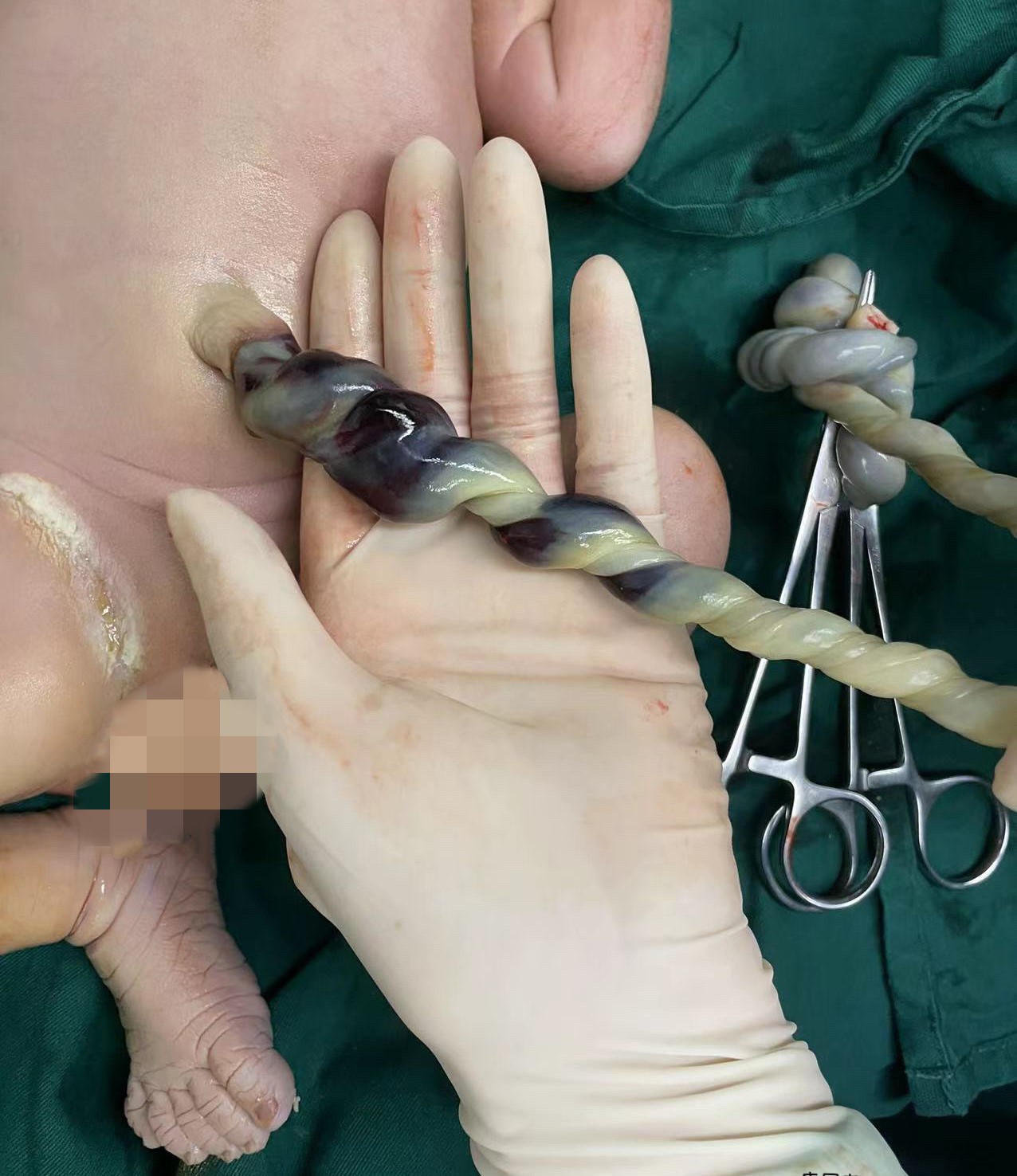

Supplement: Supplementary Figure 1 — Gross image of umbilical cord in a live fetus, showing vascular thrombosis with cord torsion. These features suggest mechanical compromise of cord blood flow. [file Image1.jpeg]

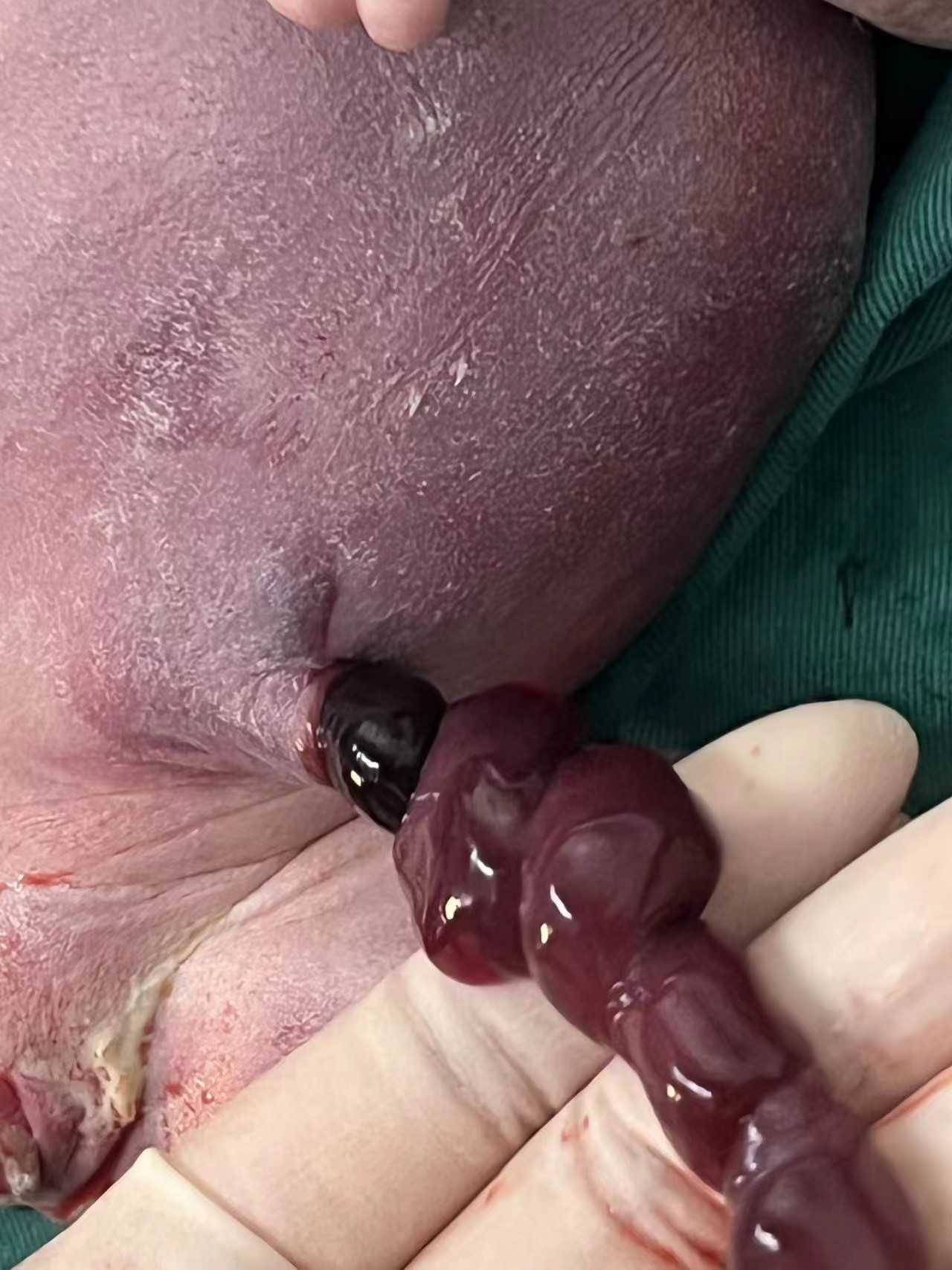

Supplement: Supplementary Figure 2 — Gross pathological specimen of a stillborn fetus showing thrombosis and torsion at the umbilical wheel. The finding supports a mechanical and thrombotic etiology for intrauterine demise. [file Image2.jpeg]

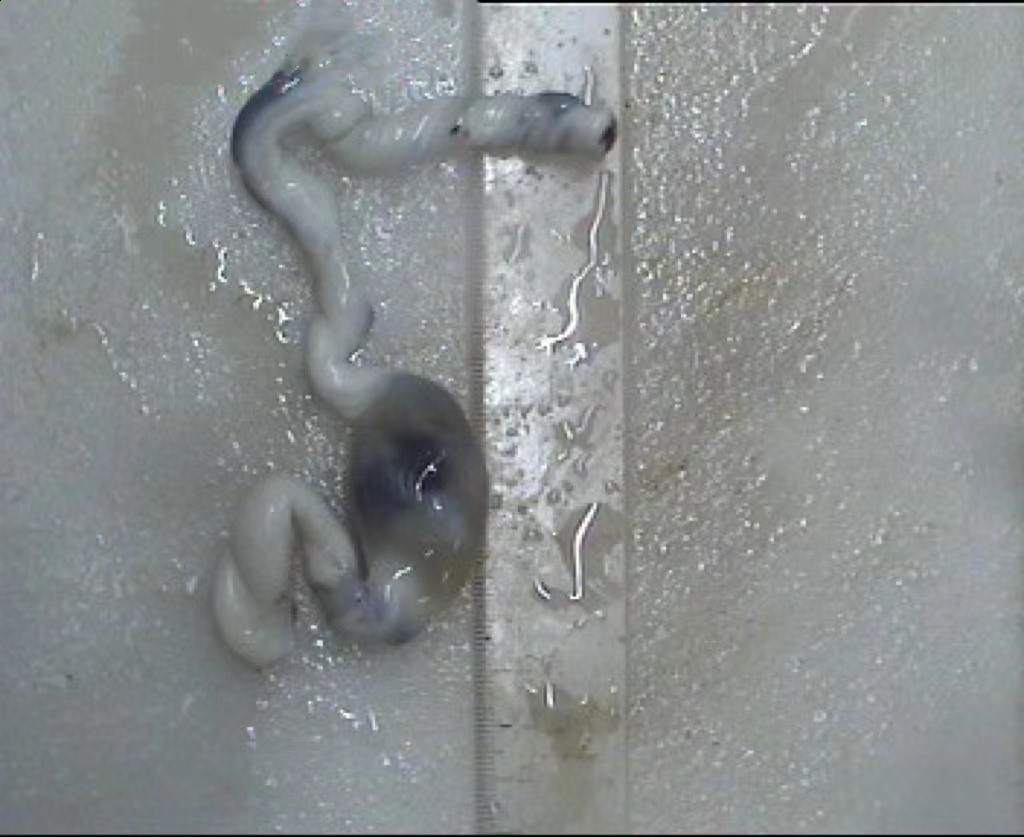

Supplement: Supplementary Figure 3 — Pathological specimen of an umbilical cord showing a prominent 2 cm × 4 cm protrusion consistent with thrombus. The mass caused focal vascular dilation and distortion. [file Image3.jpeg]

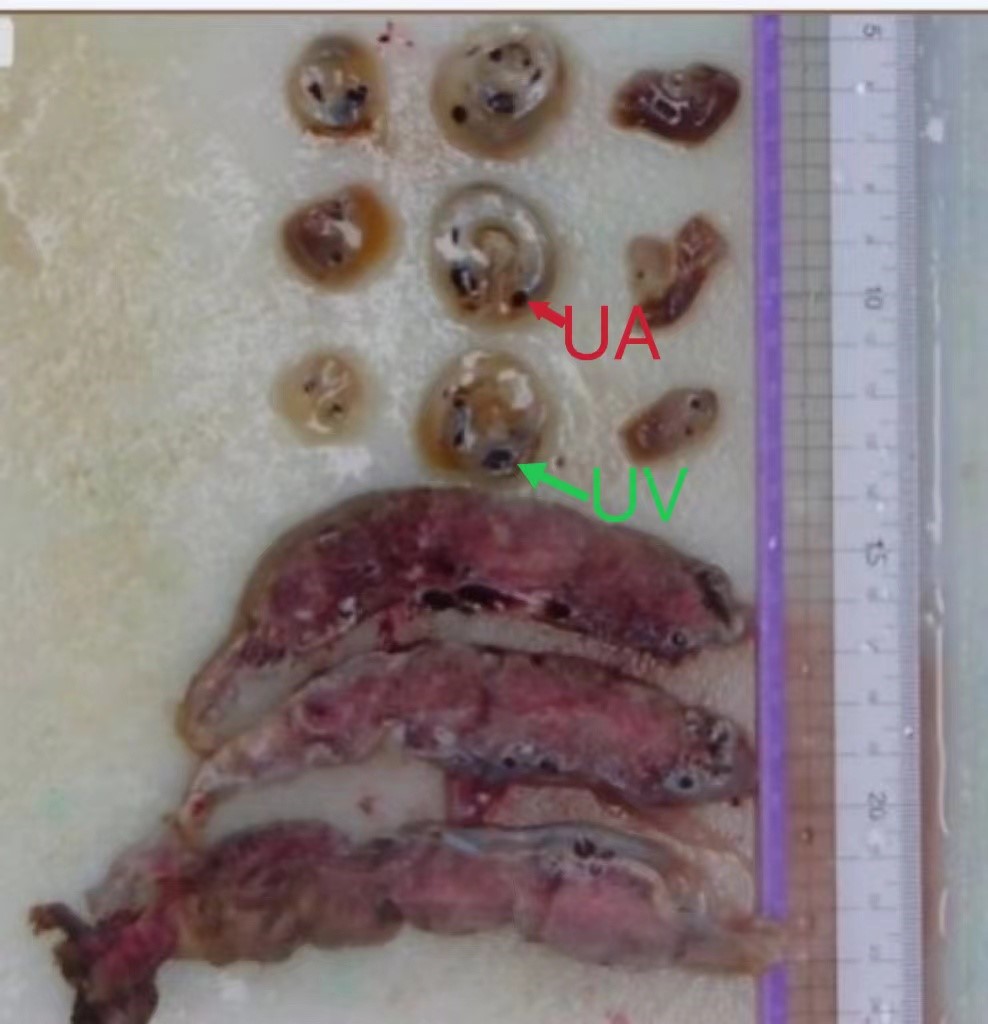

Supplement: Supplementary Figure 4 — Cross section of the umbilical cord and gross placenta specimen. Visible thrombi are seen within a single umbilical artery (UA) and the umbilical vein (UV), indicating mixed arterial and venous thrombosis. [file Image4.jpeg]

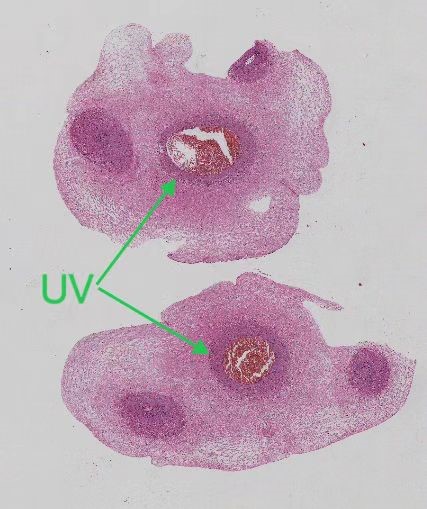

Supplement: Supplementary Figure 5 — Hematoxylin and eosin (HE) staining of umbilical cord (40× magnification) showing thrombus formation in a single UA and UV. Scale bar = 50 µm. [file Image5.jpeg]

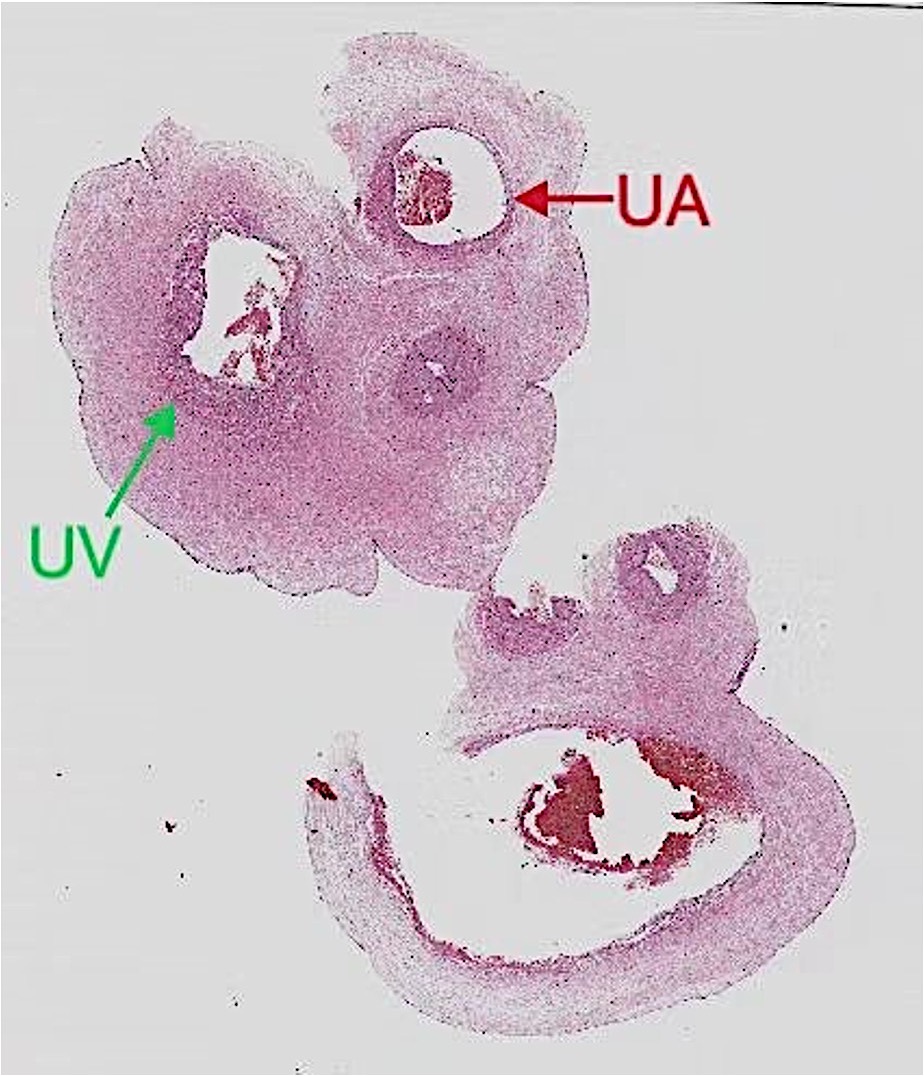

Supplement: Supplementary Figure 6 — Hematoxylin and eosin (HE) staining (40× magnification) revealing thrombus within the umbilical vein (UV). Scale bar = 50 µm. [file Image6.jpeg]
